# Transcatheter Electrosurgical Unification of a Fenestrated Atrial Septal Defect for Single Device Closure

**DOI:** 10.1016/j.jaccas.2026.108395

**Published:** 2026-05-29

**Authors:** David A. Lyons-Ewing, Cameron McAlister, Aniket Puri, Richard W. Troughton, Philip D. Adamson

**Affiliations:** a Cardiology Department, Christchurch Hospital, Te Whatu Ora–Health New Zealand, Christchurch, New Zealand; b Christchurch Heart Institute, Christchurch, New Zealand; c University of Otago, Christchurch, New Zealand

**Keywords:** atrial septal defect closure, fenestrated atrial septal defect, transcatheter electrosurgery

## Abstract

**Objectives:**

To describe the use of transcatheter electrosurgical septal tendon transection to consolidate a fenestrated atrial septal defect into a single defect suitable for single-device closure.

**Key Steps:**

1) Cross the anterior and posterior fenestrations with separate guiding catheters under transesophageal echocardiography guidance. 2) Establish through-and-through access under fluoroscopy by advancing a guidewire into the left atrium and capturing it with a snare. The guidewire in use is preprepared by removing insulation over a short segment and shaping this into a controlled “flying V” configuration before insertion, allowing it to function as the electrosurgical interface. 3) Apply controlled tension and deliver cut diathermy with nonionic flush to transect the septal tendon. 4) Balloon-size the unified defect and deploy a single septal occluder.

**Potential Pitfalls:**

Risks include thromboembolism from inadequately incorporated tissue, incomplete capture of the divided tendon, and thermal injury to adjacent structures. Continuous transesophageal echocardiography guidance and careful case selection aim to mitigate these hazards.

**Take-Home Message:**

Electrosurgical tendon transection can convert a fenestrated atrial septal defect into a single defect, enabling stable single-device percutaneous closure.

## Visual Summary

**Figure:**
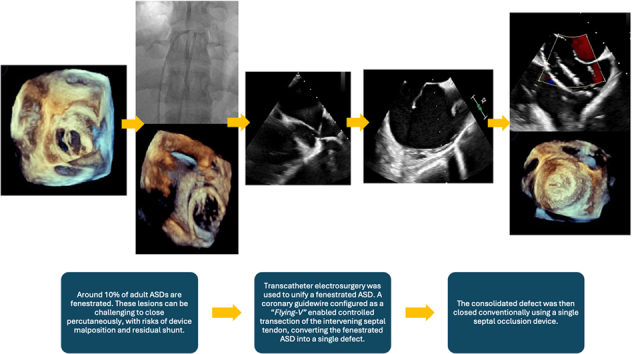

Atrial septal defects (ASDs) are the second most common congenital cardiac anomaly in adults.^1^ If left untreated they can result in significant complications, including paradoxical embolism, atrial arrythmias, right heart dysfunction, and pulmonary hypertension.^2^

**Take-Home Message**

• Transcatheter electrosurgical transection of a septal tendon can be used to convert a fenestrated atrial septal defect into a single defect, enabling secure single-device percutaneous closure.

**Equipment List:** 

Item/Device	Manufacturer (If Known)
TEE probe	Philips/GE/Siemens (varies by lab)
Femoral venous access sheaths (6 F, 12 F)	Terumo/Cordis/Cook
JR4 guiding catheter 6 F	Cordis
MPA1 guiding catheter 6 F	Cordis
AL1 guiding catheter 6 F	Cordis
AR MOD guiding catheter 6 F	Cordis
Slippy Glidewire	Terumo
Astato XS guidewire	Asahi Intecc
Finecross microcatheter 1.8 F	Terumo
En Snare retrieval device	Merit Medical
Amplatz Super Stiff 0.035-inch guidewire	Boston Scientific
Amplatzer sizing balloon	Abbott (formerly St. Jude Medical)
Emerge balloon 2.0 × 12 mm/2.5 × 12 mm	Boston Scientific
Amplatzer Trevisio delivery system 8 F	Abbott
Amplatzer Septal Occluder (30 mm)	Abbott
Z-suture closure material	Ethicon (Johnson & Johnson) or similar

TEE = transesophageal echocardiography.

Well-established indications for ASD closure include hemodynamically significant left-to-right shunt and right ventricular enlargement, as well as the already mentioned associated complications.^2^ Percutaneous device closure of secundum ASDs offers comparable efficacy to surgical repair, with the advantages of shorter hospital stays and lower complication rates.^3^^,^^4^ Transcatheter ASD closure has become the gold-standard approach for anatomically suitable defects.^5^ These devices are expandable, double-disc implants made of wire mesh and covered with durable fabric (commonly polyester or Dacron). A central waist occupies the defect while the opposing discs anchor against the septal rims.^6^

**Visual Summary:**
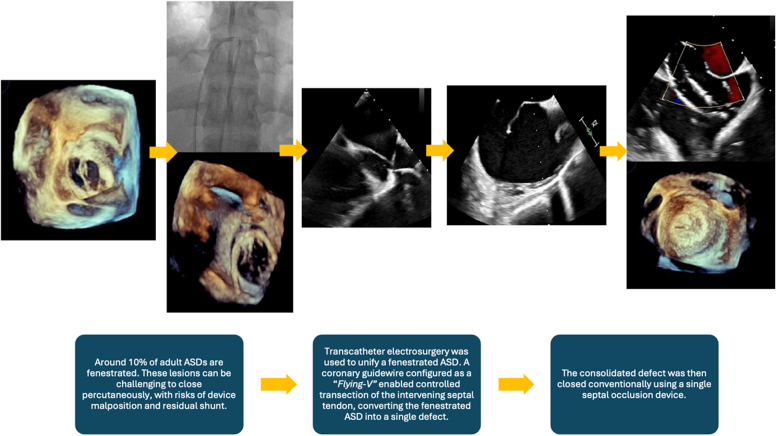
Serial Imaging Demonstrating Transcatheter Electrosurgical Unification of a Fenestrated Atrial Septal Defect for Single-Device Closure ASD = atrial septal defect.

Approximately 10% of patients present with multiple or fenestrated ASDs.^5^ When such cases are encountered, a number of strategies have been employed, including placement of multiple individual devices or use of specialized devices such as the self-centering cribriform occluder.^7^^,^^8^ Outcomes with these approaches are variable and can result in residual shunt, malposition, and device instability.^7^^,^^8^

Transcatheter electrosurgery is an emerging therapeutic modality that expands the interventionalist's toolkit for managing complex structural heart disease. Early clinical applications have demonstrated that intracardiac tissue laceration can be achieved using a partially denuded, kinked guidewire configured as a “flying-V.”^9^ This enables precise delivery of radiofrequency energy, allowing targeted tissue division.

Here we present a novel application of transcatheter electrosurgery for consolidating a fenestrated ASD into a single defect amenable to single-device closure.

## Case Summary

An otherwise healthy 42-year-old woman presented for evaluation of progressive exertional shortness of breath (NYHA functional class II). Clinical examination was significant for a grade 3/6 systolic murmur heard over the right upper sternal border. Transthoracic echocardiography demonstrated a fenestrated secundum ASD with significant left-to-right shunting. There was associated moderate biatrial enlargement and a moderately dilated right ventricle with preserved systolic function. The right ventricular systolic pressure was estimated at 29 mm Hg above right atrial pressure. The atrioventricular valves were normal.

**Figure 1:**
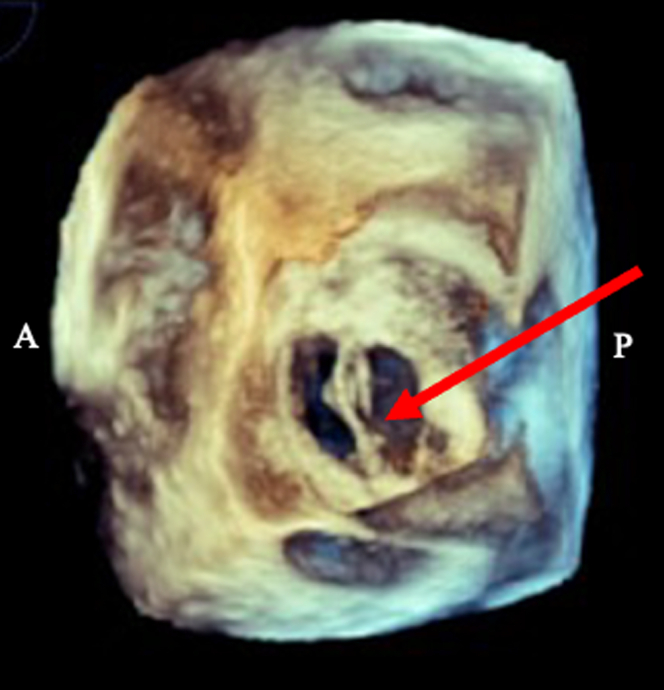
Three-Dimensional Transesophageal Echocardiography of the Fenestrated Atrial Septal Defect Three-dimensional image of the fenestrated atrial septal defect (as seen from the left atrium) demonstrating adjacent septal fenestrations and the intervening tendon ridge (red arrow). A = anterior; P = posterior.

Subsequent transesophageal echocardiography (TEE) confirmed a fenestrated interatrial septum consisting of an anterosuperior defect measuring 13 × 25 mm and a smaller posteroinferior defect measuring 8 × 24 mm separated by a tendon ridge (Figure 1). The residual septum was thin and aneurysmal. The margins were deemed amenable to transcatheter ASD closure, being at least 4 mm in diameter.

The case was reviewed at the local multidisciplinary structural heart team meeting. Given the size of the ASD, associated significant left-to-right shunt, and anticipated complications of leaving the defect untreated, a plan was made to perform percutaneous transcatheter ASD closure.

Consideration was given to traditional approaches for fenestrated ASD closure. We anticipated that deploying independent occluders into each fenestration would result in both residual shunt and device instability, particularly given the elliptical shape and proximity of the fenestrations. Because the defect had otherwise favorable anatomical parameters, we sought to consolidate the fenestrations to enable secure closure with a single occlusion device. Balloon septoplasty was considered; however, the intervening tendon was not clearly amenable to adequate mechanical deformation, raising concern that a persistent postclosure shunt would remain with this technique. In contrast, dividing the septal tendon using transcatheter electrosurgery offered a controlled method to consolidate the fenestrations into a single defect, enabling secure closure with a single occluder. Given the emerging evidence supporting electrosurgery for precise intracardiac tissue modification, this was considered a potential effective and elegant solution for this case.

## Procedural Steps

**Figure 2:**
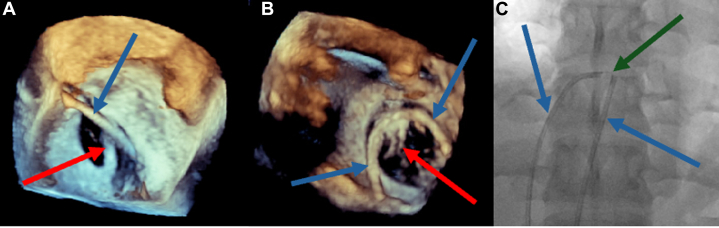
Establishing Independent Catheter Access Across Both Fenestrations and Creation of the “Flying V” Configuration Required for Targeted Tendon Transection This figure demonstrates the procedural steps used to obtain independent access across both fenestrations and to assemble the “flying-V” configuration required for controlled manipulation of the septal tendon. (A) TEE view from the left atrium. A JR4 guiding catheter (blue arrow) is advanced across one fenestration. The septal tendon is indicated by the red arrow. (B) Establishment of through-and-through access, with both JR4 guiding catheters (blue arrows) independently crossing the anterior and posterior fenestrations. The Astato XS coronary wire is captured within the left atrium using the En Snare device. (C) Fluoroscopic image illustrating the “flying-V” configuration, with both guiding catheters (blue arrows) and the exposed segment of the coronary wire forming the active interface for targeted tendon transection (green arrow). TEE = transesophageal echocardiography.
• The working segment of the Astato XS wire had been prepared so that a short segment of the insulation was removed over several millimeters, exposing a controlled portion of metal. This exposed segment was shaped so that it lay along the inner curvature of a deliberate bend, creating a “flying-V” profile (Figure 2C). With both limbs of the wire insulated within their respective catheters, only the intended inner surface of the bend functioned as the active interface.
• With the septal tendon maintained under controlled tension (Figure 3A), the prepared segment enabled targeted transection of the tendon using 50 W of cut diathermy (Figure 3B). A nonionic flush (5% dextrose) was used to displace blood and improve charge concentration at the tissue while reducing coagulation.

After informed consent was obtained, the patient underwent general anesthesia and endotracheal intubation. We performed the following steps under fluoroscopic and TEE guidance:

• Femoral venous access was achieved under ultrasound guidance. Two venous punctures allowed placement of 6-F and 12-F sheaths to facilitate device delivery and adjunctive catheter manipulation.
• An initial attempt at balloon septoplasty was performed using an Amplatzer sizing balloon to mechanically deform the intervening septal tendon. This resulted in a residual crescentic defect with persistent shunting, indicating that adequate unification of the fenestrations could not be achieved with this technique.
• The anterior and posterior fenestrations of the atrial septal defect were each crossed independently using separate JR4 guiding catheters (Figure 2A). Through one JR4 catheter, an Astato XS coronary wire was directed across the selected fenestration and advanced into the left atrium. In parallel, an En Snare device was advanced via the second JR4 catheter and used to capture the Astato wire within the left atrium. This maneuver established through-and-through access across the tendon ridge via both fenestrations (Figure 2B).

**Figure 3:**
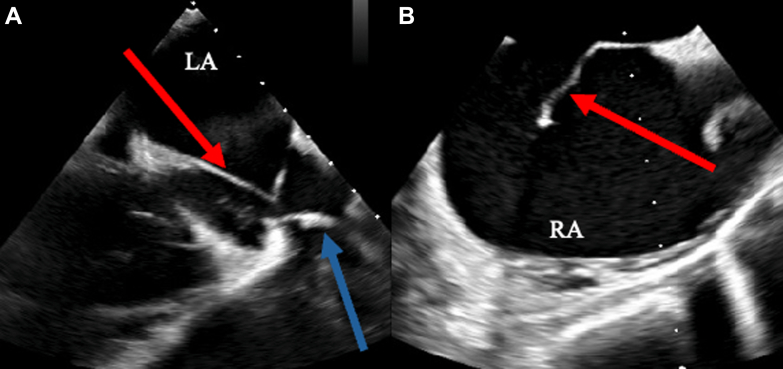
Controlled Manipulation and Transection of the Septal Tendon This figure illustrates the key procedural steps involved in tensioning and dividing the septal tendon to unify the adjacent fenestrations. (A) The septal tendon is placed under controlled tension (red arrow) using the established through-and-through wire system. The guiding catheters carrying the exposed working segment of the prepared coronary wire (blue arrow) are positioned so that the active inner curvature of the “flying-V” configuration lies directly against the tendon, enabling precise energy delivery. (B) After application of cut diathermy, the tendon is fully transected (red arrow). LA = left atrium; RA = right atrium.
• This effectively unified the 2 adjacent fenestrations into a single larger defect suitable for conventional, single-device closure.
• The resulting defect was measured using a 34-mm Amplatzer sizing balloon (Figure 4A). The stretched diameter confirmed suitability for closure with a 30-mm occlusion device.

**Figure 4:**
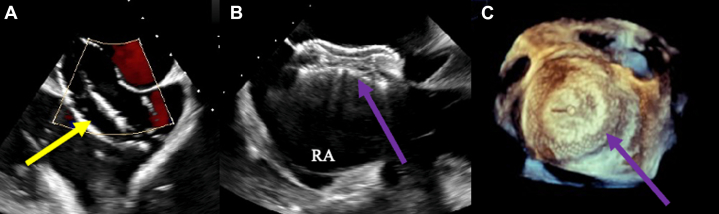
Assessment of the Unified Defect and Confirmation of Septal Occluder Position This figure demonstrates evaluation of the unified defect created after tendon transection and confirmation of final device position. (A) The unified defect is measured using a sizing balloon (yellow arrow) to determine the stretched diameter and guide device selection. (B) TEE confirms appropriate positioning of the septal occluder (purple arrow) and is used to verify that the divided septal tendon is incorporated within the device waist. (C) Three-dimensional TEE provides an en face view of the occluder (purple arrow), showing complete coverage of the defect and stable integration within the septal anatomy. RA = right atrium; TEE = transesophageal echocardiography.
• An 8-F Amplatzer Trevisio delivery system was advanced via the 12-F femoral sheath. A 30-mm Amplatzer Septal Occluder was deployed across the defect incorporating the free portion of the divided tendon ridge. TEE confirmed that all rims of the defect were adequately captured (Figures 4B and 4C). A push-pull test confirmed device stability and exclusion of residual shunt.
• At the conclusion of the procedure, all catheters and sheaths were removed, and hemostasis was achieved using a Z-suture at the femoral access site.

The procedure was well tolerated without complications, with a total duration of 1 hour.

Midterm follow-up at 10 months demonstrated a stable septal occluder on transthoracic echocardiography, with no evidence of residual shunt on agitated-saline contrast imaging.

## Potential Pitfalls

Potential complications include thromboembolism arising from inadequately incorporated transected tissue or incomplete capture of the divided septal tendon within the device waist. Thermal injury to adjacent structures is another recognized risk when applying transcatheter electrosurgery. Defects in close proximity to critical anatomical structures may therefore be unsuitable for electrosurgical modification. A further conceivable pitfall is that intervening septal tendons may contribute to the structural integrity and geometry of fenestrated defects; their division may alter the aperture and morphology in ways that may affect subsequent device stability or the anticipated closure strategy. Careful case selection and continuous intraprocedural TEE guidance are essential to identify these hazards early and minimize their impact.

## Conclusions

We have demonstrated that transcatheter electrosurgical septal tendon transection is an effective way of consolidating a fenestrated ASD into a single defect. This allows subsequent closure with a conventional septal occlusion device. The technique we describe offers the interventional cardiologist a valuable option when managing fenestrated ASDs that are unlikely to be satisfactorily treated with independent devices because of overlap, inadequate circumferential rim apposition, and the associated risk of residual shunt. Patients in whom a unified defect is anticipated to yield stable, securely captured single-device closure represent the most appropriate candidates for this approach.

## Funding Support and Author Disclosures

The authors have reported that they have no relationships relevant to the contents of this paper to disclose.
